# Serum proteomics links the cardiorespiratory biomarkers CTRC, OSM, and MMP-10 to exacerbation severity and number in patients with COPD

**DOI:** 10.1042/CS20255852

**Published:** 2025-05-09

**Authors:** Eduardo I. Cardenas, Kristina Andelid, Nikolaos Pournaras, Ann Ekberg- Jansson, Nicola Orsini, Georgios Stratelis, Tomas Jernberg, Anders Lindén

**Affiliations:** 1 Division of Lung and Airway Research, Institute of Environmental Medicine, Karolinska Institutet, Stockholm, Sweden; 2 COPD Center, Department of Internal Medicine and Clinical Nutrition, Institute of Medicine, Sahlgrenska Academy, University of Gothenburg, Gothenburg, Sweden; 3 COPD Center, Department of Respiratory Medicine and Allergology, Sahlgrenska University Hospital, Gothenburg, Sweden; 4 Karolinska Severe COPD Center, Department of Respiratory Medicine and Allergy, Center for Molecular Medicine, Karolinska University Hospital, Stockholm, Sweden; 5 Department of Research and Development, Region Västra Götaland, Gothenburg, Sweden; 6 Department of Public Health Sciences, Karolinska Institutet, Stockholm, Sweden; 7 AstraZeneca Nordic, Stockholm, Sweden; 8 Department of Medical Sciences, Respiratory, Allergy and Sleep Research, Uppsala University, Uppsala, Sweden; 9 Department of Clinical Sciences, Danderyd Hospital, Karolinska Institutet, Stockholm, Sweden

**Keywords:** COPD, CTRC, eosinophil, exacerbation, MMP-10, OSM, proteomics

## Abstract

During exacerbations, patients with chronic obstructive pulmonary disease (COPD) are at risk for severe cardiovascular disease (CVD). Despite this, the available literature on systemic biomarkers of CVD during exacerbations is limited. In the present study, a proteomic approach was used to assess alterations in the concentrations of 177 biomarkers of CVD and inflammation in serum samples from 26 long-term smokers (LTS) with mild-to-severe COPD (GOLD stage 1–3) and chronic bronchitis (COPD-CB) but no allergy. These patients were followed for 60 weeks, and they all provided paired samples during stable disease and exacerbations. Serum samples from ten healthy non-smokers (HNS) and ten LTS without COPD or CB constituted controls. Of all the proteins analyzed, only chymotrypsin C (CTRC), oncostatin M (OSM), and matrix metalloproteinase 10 (MMP-10) displayed significantly altered concentrations during exacerbations in the COPD-CB group. Here, the concentrations of CTRC and OSM correlated with exacerbation severity, CRP, blood leukocytes, and other cardiovascular biomarkers. In contrast, the concentration of MMP-10 during stable disease correlated with blood eosinophil counts and exacerbation numbers. Finally, the concentrations of OSM and MMP-10 during stable disease correlated with blood leukocytes and tobacco load, respectively. Our study suggests that CTRC, OSM, and MMP-10 bear potential as cardiorespiratory biomarkers in patients with COPD and CB. Collectively, these biomarkers display substantial alterations during exacerbations and correlate with the severity and number of exacerbations. These results motivate prospective studies to determine the clinical utility of CTRC, OSM, and MMP-10 in assessing cardiorespiratory risk in patients with COPD.

## Introduction

Chronic obstructive pulmonary disease (COPD) is a common lung disorder characterized by chronic airflow limitation, local and systemic inflammation, poor quality of life, and high mortality [1]. A key risk factor for COPD is long-term tobacco smoking, and the typical inflammatory profile associated with COPD encompasses local accumulation of cytotoxic T cells, alveolar macrophages, and neutrophils, as well as a systemic increase in circulating neutrophils [1]. Unfortunately, the precise mechanisms driving COPD are still poorly understood, and the available pharmacological therapies can only reduce symptoms and risk of progression or death in a modest manner. Given that exacerbations of COPD constitute a strong risk factor for poor prognosis, it seems feasible that further study of this complication may shed light on key pathogenic mechanisms [2].

Historically, research on COPD has centered on two common pulmonary comorbidities: emphysema and chronic bronchitis (CB). Nevertheless, a growing body of literature now indicates that cardiovascular disease (CVD) also plays an important role in the morbidity and mortality of patients with COPD. In fact, CVD is up to five times more likely to occur in patients with COPD compared with the general population [3], and CVD is a common cause of death among these patients [4]. Furthermore, the risk to experience and die of CVD may be enhanced up to four times during exacerbations in patients with COPD [5].

Given the large number of patients with COPD and the limited clinical resources available, numerous attempts have been made to identify easily accessed biomarkers that can help define and prioritize patients with a high risk for poor prognosis. Along these lines, C-reactive protein (CRP) in blood has been used to assess systemic inflammation in COPD [6], while several cytokines involved in neutrophil mobilization (e.g. IL-6, IL-8, and IL-17) have been used as local biomarkers of disease severity and risk for CVD in COPD patients [7–11]. Nevertheless, most biomarkers have been characterized in patients with stable COPD, who are easier to study, while the literature on biomarkers for exacerbations of COPD remains limited. To date, fibrinogen in plasma emerges as one of the best-documented protein biomarkers for the risk of exacerbations in COPD patients [12–14]. However, a single biomarker is unlikely to reflect all important aspects of COPD pathology, and the interpretation of a single protein in any body fluid can be confounded by common inflammatory conditions beyond COPD. Therefore, we think that there is a strong rationale for new studies aiming to identify novel biomarker signatures for critical aspects of COPD, including exacerbations and the risk of CVD.

In the present study, we aimed to identify systemic alterations in biomarkers of CVD during COPD exacerbations, using biomarkers of inflammation as a reference. For this purpose, we used a proteomic approach to assess 177 biomarkers in serum samples from a group of long-term smokers (LTS) with COPD and CB (COPD-CB). In this group, we performed longitudinal comparisons between paired samples collected during stable disease and an exacerbation, respectively. In addition, we performed cross-sectional comparisons between this COPD-CB group during stable disease and two control groups: clinically healthy non-smokers (HNS) and LTS without COPD and CB.

## Methods

### Patient material

The patient material used in this study derives from a larger material that has previously been described in detail [9,15,16]. In brief, LTS with COPD-CB were recruited via advertisement and from the outpatient clinic of the Department of Respiratory Medicine of the Sahlgrenska Hospital, Gothenburg, Sweden. During the inclusion visit, we confirmed that all LTS with COPD-CB had an FEV1/FVC ratio of less than 0.7 (COPD) and had experienced a productive cough for at least three months per year for two consecutive years (CB). The exclusion criteria encompassed the following: (I) any additional lung diseases other than COPD-CB; (II) allergic sensitization to one or more common aeroallergens (Phadiatop^TM^); (III) regular use of oral glucocorticoids; (IV) long-term oxygen therapy; (V) drug abuse; (VI) cancer; (VII) severe mental illness besides well-treated depression; (VIII) chronic inflammatory diseases such as rheumatoid arthritis and inflammatory bowel disease; and (IX) congestive heart failure or coronary artery disease. The aeroallergens screened for were plant pollens (birch, timothy grass, mugwort, olive, and pellitory), pet dander (dog, cat, and horse), house dust mite, and *Cladosporium* (mold). Serum samples were collected during an inclusion visit and every 15th week for 60 weeks. In addition, serum and sputum samples were collected whenever a patient with COPD and CB experienced an acute exacerbation, which was diagnosed according to established criteria [17,18]. In brief, patients experiencing dyspnoea and/or cough and sputum worsening less than 14 days were judged as having an exacerbation. This exacerbation sample was collected prior to emergency treatment. Moreover, a group of HNS and a group of LTS without COPD and CB were also recruited and used as control groups. All participants underwent a urine cotinine test to confirm smoking status, and long-term smoking was defined as more than 10 pack-years.

In the current study, we analysed paired serum samples from LTS with COPD-CB collected during a stable COPD visit and an exacerbation visit, respectively. The majority of samples from stable COPD (20 out of 26) were collected during the inclusion visit. Because six included patients experienced an exacerbation during their inclusion visit, their matched samples from stable disease were harvested 15 weeks after their exacerbation pair. Finally, all LTS with COPD-CB included in this study experienced at least one exacerbation during the course of the study, and all exacerbation samples used in this study correspond to the first exacerbation of each patient. Exacerbations were classified as mild, moderate, or severe according to GOLD criteria [1]. In brief, mild exacerbations only required treatment with short-acting bronchodilators in the outpatient clinic, moderate exacerbations required treatment with oral glucocorticoids and/or antibiotics in the outpatient clinic, and severe exacerbations required hospitalization. Importantly, no patients included in this study experienced pneumonia during their inclusion visit, exacerbation visit, or in between these two visits.

### Serum proteomics

Commercially available panels (Olink^®^ Target 96 Cardiovascular II and Inflammation panels) were used to measure protein biomarkers in serum samples by proximity extension assay [19] at the facilities of Olink^®^ proteomics (Uppsala, Sweden). In this method, two antibodies labeled with complementary DNA oligonucleotides bind a target protein in solution, and the close proximity allows the hybridization of the DNA oligonucleotides. The hybridized DNA labels are then extended by a DNA polymerase to generate a unique DNA barcode that can be amplified and detected via next-generation sequencing (NGS). In brief, this high-throughput method combines the specificity of antibody-based immunoassays with the powerful amplification potential of polymerase chain reaction (PCR) and next-generation sequencing (NGS). Each panel detected 92 proteins (Supplementary Tables 1 and 2) and there were seven proteins that overlapped between the two panels for a total of 177 unique proteins. Results are reported as normalized protein expression (NPX), an arbitrary unit in the log base 2 scale.

### Randomization and blinding

Samples were randomly assigned to wells in 96-well plates before delivery to Olink^®^ proteomics for analysis. Notably, each sample was assigned a codename before analysis to guarantee blind assessment.

### Statistical analysis

Paired and unpaired comparisons between the two groups were assessed by paired and unpaired Student’s *t*-test, respectively. Multiple comparisons were performed via ANOVA followed by Tukey’s post hoc test to compare each group against the others. Correlations were determined by Spearman’s rank test. *P* values were corrected for multiple testing using the Benjamini–Hochberg method. Statistical analyses of proteomics data were done using Python 3·10·9, while all other statistical analyses were performed in Prism 9.3 (GraphPad).

## Results

### Patient characteristics

The main study group consisted of 26 LTS with COPD and CB (COPD-CB group), while the two control groups consisted of 10 HNS and 10 LTS without COPD and CB, respectively. The main characteristics of all study participants during stable clinical conditions are summarized in Table 1 (see also “Methods” for more information). Although the median age was lower in the LTS control group when compared with the other groups (LTS vs COPD-CB: *P*=0.03 & LTS vs HNS: *P*=0.002 determined by Kruskal–Wallis test followed by Dunn’s multiple comparison test), the median number of pack-years was similar between the LTS group and the COPD-CB group (*P*=0.09 determined by Mann-Whitney test). In addition, the main clinical characteristics of LTS with COPD-CB during exacerbation are summarized in Table 2. In general, most exacerbations included in the present study (20 out of 26) were moderate, and only one exacerbation required hospitalization.

**Table 1:** Clinical characteristics of study groups during stable clinical conditions.

	HNS (*n* = 10)	LTS (*n* = 10)	LTS with COPD-CB (*n* = 26)
Females/males (n)	7/3	8/2	14/12
Age (years)	67.5 (47–70)	50 (26–64)	62 (45–74)
Historic smoking (pack-years)	N/A	29 (13–43)	39.5 (14–80)
FEV_1_ (% of predicted)	120 (97–137)	106 (83–119)	54 (29–86)
FEV_1_/FVC ratio	0.79 (0.73–0.84)	0.77 (0.73–0.84)	0.55 (0.29–0.69)
GOLD stage (n)	N/A	N/A	I: 2; II: 13; III: 9; IV**:** 2
DLCO (% of predicted)	98 (75–139)	87 (77–111)	70 (44–100)
Cardiovascular comorbidities	N/A	N/A	Hypertension: 5 Hypercholesterolemia: 4 Sinus tachycardia: 1
CRP (mg/dL)	0.76 (0.2–5.7)	0.74 (0.23–5.8)	2.25 (0.3–15)
Leukocytes ( × 10^9^ /L)	4.95 (4.0–7.6)	7.45 (4.2–12.5)	6.8 (5.1–13.5)
Neutrophils ( × 10^9^ /L)	2.75 (1.8–4.7)	4.4 (2.5–7.8)	4.05 (2.8–9.6)
Lymphocytes ( × 10^9^ /L)	1.6 (1.3–2.6)	2.35 (1.0–3.7)	2.0 (1.3–3.4)
Monocytes ( × 10^9^ /L)	0.4 (0.05–0.7)	0.45 (0.3–0.9)	0.6 (0.3–0.9)
Eosinophils ( × 10^9^ /L)	0.1 (0.0–0.4)	0.1 (0.07–0.3)	0.1 (0.0–0.5)
Basophils ( × 10^9^ /L)	0.0 (0.0–0.1)	0.0 (0.0–0.1)	0.0 (0.0–0.1)

All data are presented as median (range), unless indicated otherwise. One patient had both hypertension and hypercholesterolemia. Data on CRP and leukocytes were only available for 20 out of 26 LTS with COPD-CB.

HNS, healthy non-smokers; LTS, long-term smokers without COPD and CB; COPD, chronic obstructive pulmonary disease; CB, chronic bronchitis; FEV_1_, forced expiratory volume in one second; FVC, forced vital capacity; GOLD, global initiative for chronic obstructive lung disease; DLCO, diffusion capacity of the lungs for carbon monoxide; CRP, C-reactive protein; NA, not applicable.

**Table 2:** Clinical characteristics of LTS with COPD-CB during exacerbation.

	**LTS with COPD-CB (*****n*** **=** **26)**
Exacerbation severity	Mild: 5 Moderate: 20 Severe**:** 1
Sputum cultures	*Haemophilus influenzae*: 9 *Haemophilus parainfluenzae*: 5 *Streptococcus pneumoniae*: 3 *Moraxella catarrhalis*: 1 Normal oropharyngeal flora**:** 6
CRP (mg/dL)	6.9 (1.1–210)
Leukocytes ( ×10^9^ /L)	9.1 (6.5–15)
Neutrophils ( × 10^9^ /L)	6.1 (3.7–10)
Lymphocytes ( × 10^9^ /L)	2.0 (1.1–3.1)
Monocytes ( × 10^9^ /L)	0.8 (0–1.4)
Eosinophils ( × 10^9^ /L)	0.1 (0–0.6)
NE (µg/mL)	54.1 (28.5–91.8)
MPO (µg/mL)	33.6 (21.4–57)

All data are presented as median (range) unless indicated otherwise. The sputum culture of one patient was positive for both *M. catarrhalis* and *H. parainfluenzae,* while the sputum culture of another patient was positive for both *H. influenzae* and *H. parainfluenzae*. Four patients did not provide sputum samples during exacerbation. Data on CRP and leukocytes were available for 20 out of 26 patients. Data on NE and MPO were available for 16 and 19 out of 26 patients, respectively.

LTS, long-term smokers; COPD, chronic obstructive pulmonary disease; CB, chronic bronchitis; CRP, C-reactive protein; NE, neutrophil elastase; MPO, myeloperoxidase.

### Altered serum concentrations of CTRC, OSM, and MMP-10 during exacerbation

**Figure 1:**
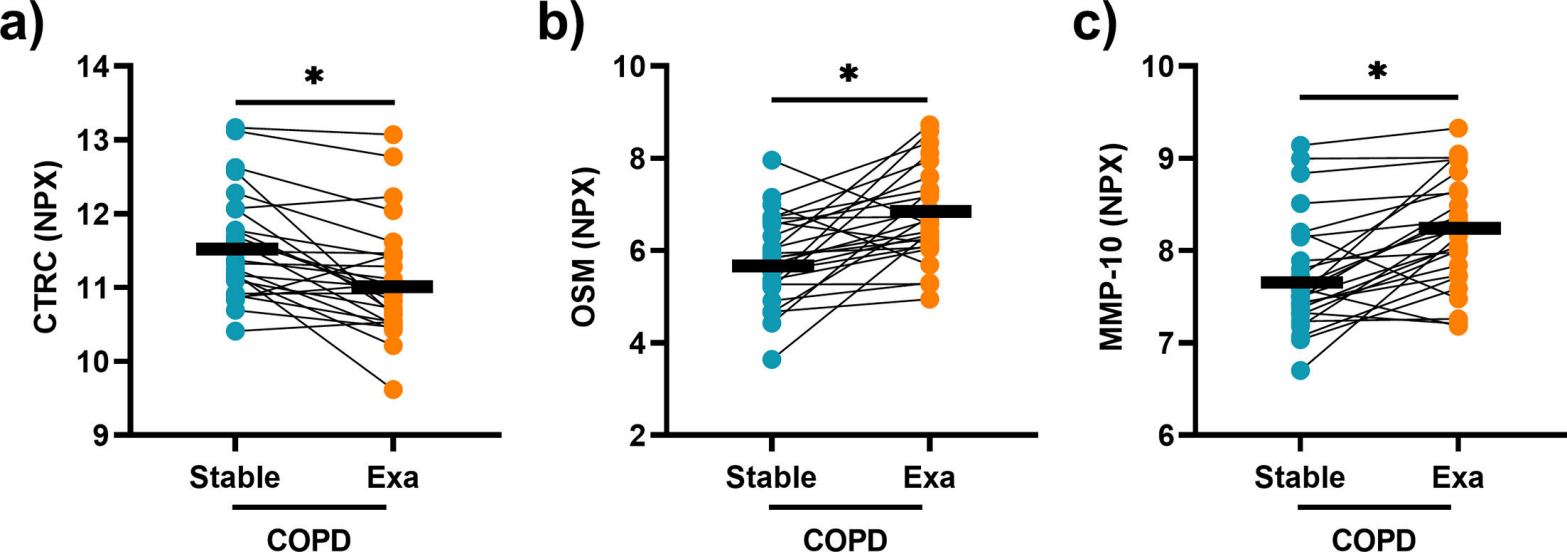
The serum concentrations of CTRC, OSM, and MMP-10 are altered during exacerbation in patients with COPD and CB. Concentrations of (**a**) CTRC, **(b**) OSM, and (**c**) MMP-10 quantified in paired serum samples from each patient during stable disease and exacerbation (Exa), respectively. Circles joined by a line represent individual patients (*n* = 26). Mean values are indicated by bold horizontal lines. Statistical significance was determined by paired Student’s t-test and *P* values were adjusted for multiple testing using the Benjamini-Hochberg method. *adj. *P*<0·05.

We found that the serum concentration of chymotrypsin C (CTRC) was decreased, while the corresponding concentrations of oncostatin M (OSM) and matrix metalloproteinase 10 (MMP-10) were increased, during exacerbation in the COPD-CB group (Figure 1A–C). We did not detect any statistically significant alteration in the serum concentrations of the other 174 proteins investigated during exacerbation. Notably, the serum concentrations of CTRC, OSM, and MMP-10 during stable disease in the COPD-CB group resembled those of the HNS and LTS control groups (Supplementary Figure S1). Furthermore, we did not detect any sex-related differences in the serum concentrations of CTRC, OSM, and MMP-10 (Supplementary Figure S2), and we found that having cardiovascular comorbidities at the time of inclusion did not have an impact on the serum concentrations of these three biomarkers (Supplementary Figure S3).

### Lower serum concentration of CTRC during exacerbation is associated with the presence of pathogenic bacteria in sputum cultures

**Figure 2:**
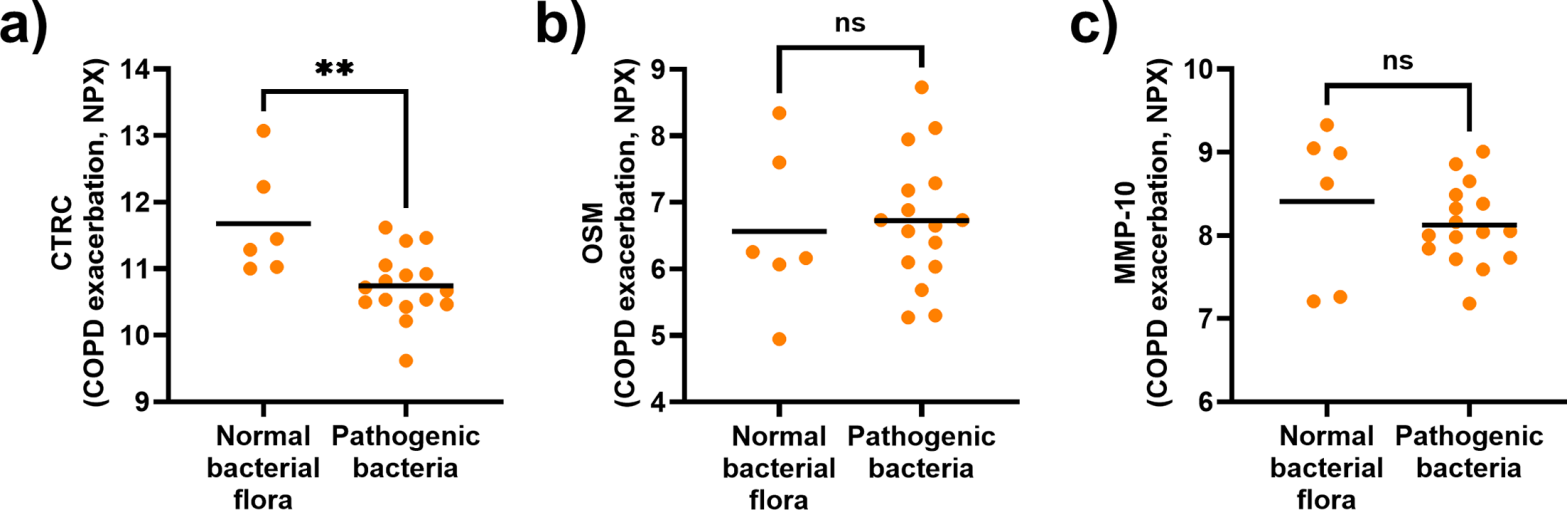
The serum concentration of CTRC, but not OSM or MMP-10, is lower in patients with COPD-CB and bacteria-induced exacerbations. Concentrations of (**a**) CTRC, (**b**) OSM, and (**c**) MMP-10 quantified in serum samples from COPD-CB patients during exacerbation with normal bacterial flora or pathogenic bacteria in sputum cultures. Closed circles represent individual patients (*n* = 22). Mean values are indicated by bold horizontal lines. Statistical significance was determined by unpaired Student’s *t*-test. **P < 0.01; ns = *P* ≥ 0.05.

Given that respiratory infections are a major cause of COPD exacerbations, we determined whether having pathogenic bacteria in sputum cultures obtained during exacerbations influenced the serum concentrations of CTRC, OSM, and MMP-10. Notably, patients with pathogenic bacteria in sputum cultures had a lower serum concentration of CTRC during exacerbations than patients without these bacteria (Figure 2A). In contrast, the serum concentrations of OSM and MMP-10 during exacerbation were similar between patients with and without pathogenic bacteria in sputum cultures (Figure 2B and C).

### Lower serum concentration of CTRC and higher serum concentration of OSM are associated with reduced blood oxygen during exacerbation

**Figure 3:**
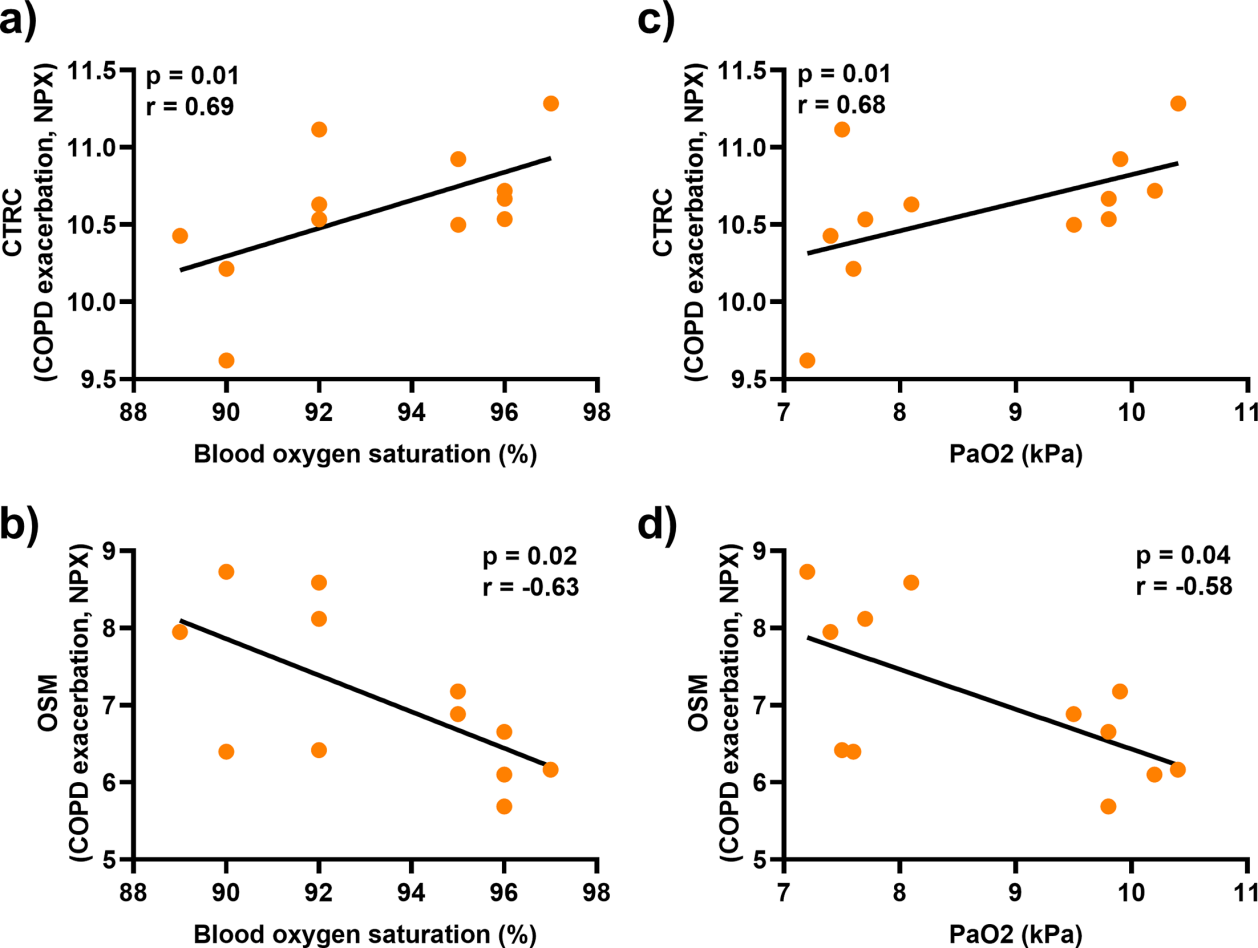
Associations of CTRC and OSM with relative arterial oxygen saturation and partial pressure of oxygen during exacerbations in patients with COPD and CB. Spearman correlation analyses of the protein levels of (**a, c**) CTRC and (**b, d**) OSM in serum and (**a-b**) the relative arterial oxygen saturation and (**c-d**) the arterial partial pressure of oxygen during exacerbation. closed circles represent individual patients (*n* = 12).

We analysed arterial blood oxygen in all patients of the COPD-CB group who had a transcutaneous oxygen saturation below 94% during exacerbation. Interestingly, CTRC and OSM displayed strong positive and negative correlations, respectively, with the relative arterial oxygen saturation (Figure 3A and B) and with the arterial partial pressure of oxygen (Figure 3C and D). However, MMP-10 did not correlate with either of those parameters (Supplementary Figure S4).

### Lower serum concentrations of CTRC and higher serum concentrations of OSM are associated with increased concentrations of CRP, leukocytes, and neutrophil-derived proteins in blood during exacerbation.

**Figure 4:**
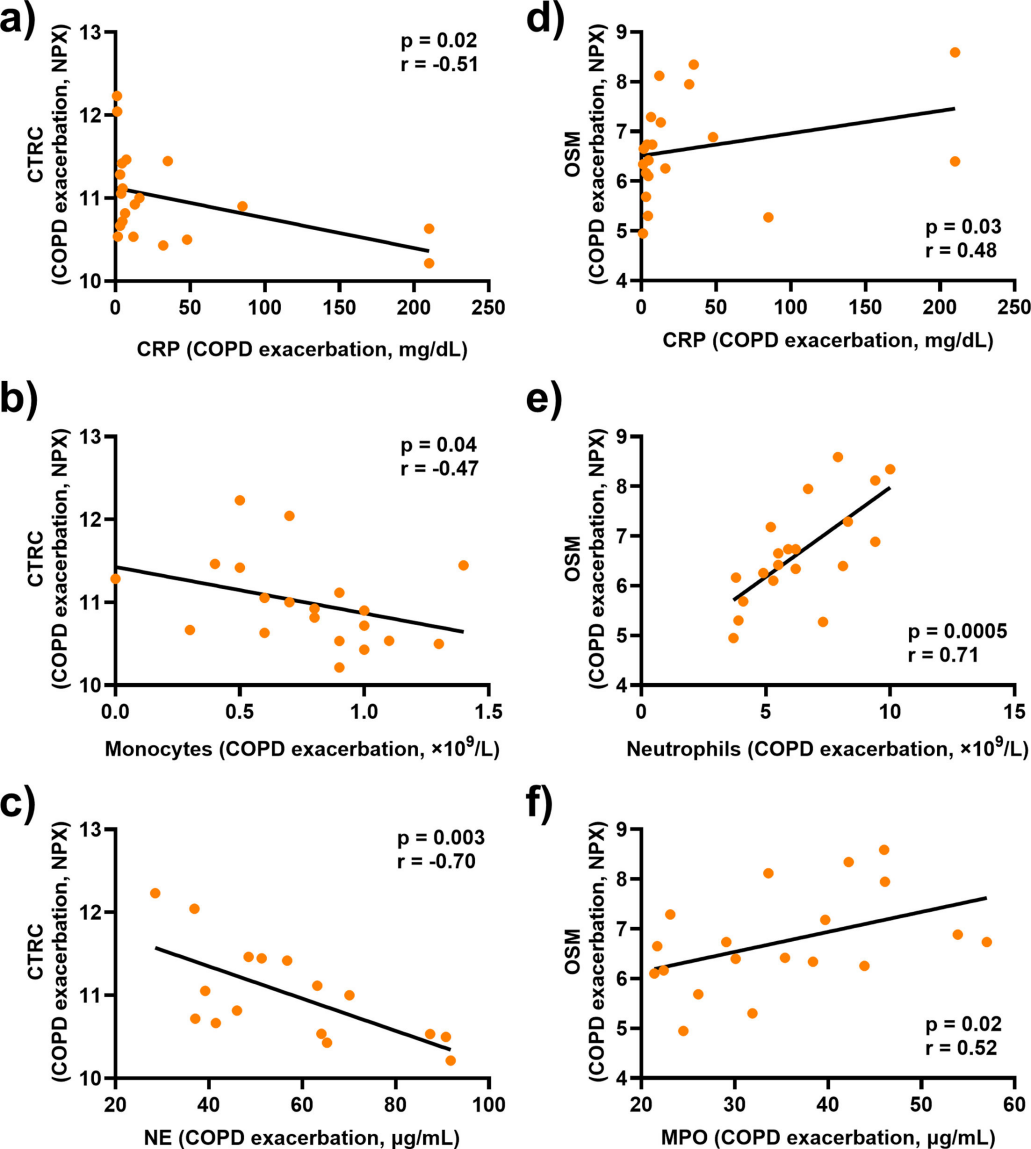
Associations of CTRC and OSM with CRP, leukocytes, and neutrophil-derived enzymes during exacerbations in patients with COPD and CB. Spearman correlation analyses of the protein levels of (**a, b, c**) CTRC and (**d, e, f**) OSM in serum and (**a**, **d**) CRP, (**b**) monocytes, (**c**) neutrophil elastase (NE), (**e**) neutrophils, and (**f**) myeloperoxidase (MPO) during exacerbation. Closed circles represent individual patients (*n* = 20 in a, b, d and e; *n* = 16 in c; *n* = 19 in f).

To further characterize the clinical relevance of CTRC, OSM, and MMP-10 during exacerbation, we assessed the relationship between these proteins and the blood concentrations of CRP and leukocytes. On one hand, a decrease in the serum concentration of CTRC during exacerbation is associated with higher blood concentrations of CRP, monocytes, and neutrophil elastase (Figure 4A–C). On the other hand, an increase in the serum concentration of OSM during exacerbation is associated with higher blood concentrations of CRP, neutrophils, and myeloperoxidase (Figure 4D–F). Notably, MMP-10 did not correlate with any of the biochemical parameters summarized in Table 2 (not shown).

### Serum concentrations of CTRC and OSM display strong correlations with those of other cardiovascular biomarkers during exacerbations.

To further characterize the clinical relevance of CTRC, OSM, and MMP-10 during exacerbation, we assessed the relationship between these proteins and the blood concentrations of CRP and leukocytes. On one hand, a decrease in the serum concentration of CTRC during exacerbation is associated with higher blood concentrations of CRP, monocytes, and neutrophil elastase (Figure 4A–C). On the other hand, an increase in the serum concentration of OSM during exacerbation is associated with higher blood concentrations of CRP, neutrophils, and myeloperoxidase (Figure 4D–F). Notably, MMP-10 did not correlate with any of the biochemical parameters summarized in Table 2 (not shown). Serum concentrations of CTRC and OSM display strong correlations with those of other cardiovascular biomarkers during exacerbations.

**Figure 5:**
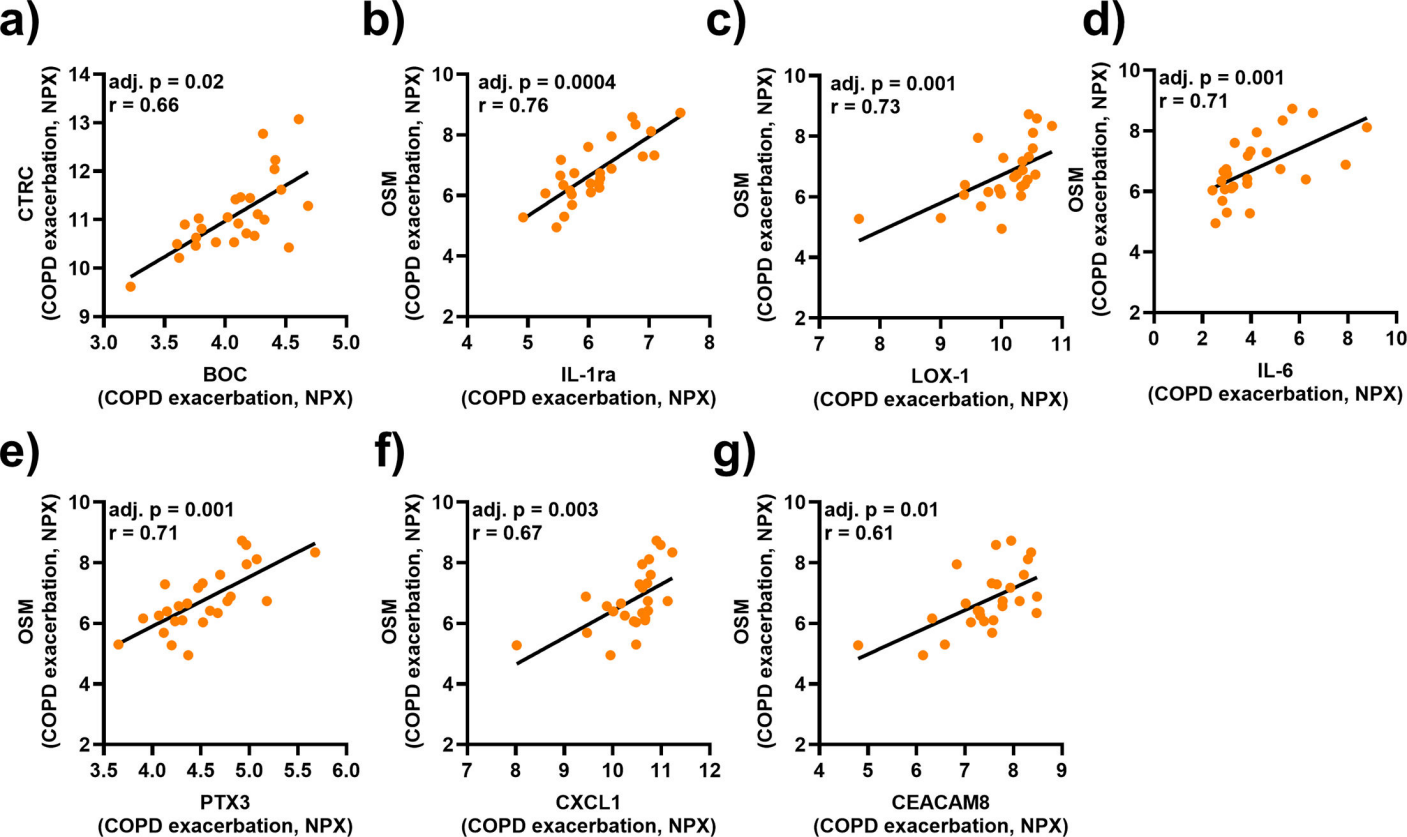
Associations of CTRC and OSM with various cardiovascular biomarkers during exacerbations in patients with COPD and CB. Spearman correlation analyses of the protein levels of (**a**) CTRC and (**b-g**) OSM in serum and (**a**) BOC, (**b**) IL-1ra, (**c**) LOX-1, (**d**) IL-6, (**e**) PTX3, (**f**) CXCL1, and (**g**) CEACAM8 during exacerbation. Closed circles represent individual patients (*n* = 26). P-values were adjusted for multiple testing using the Benjamini-Hochberg method.

We investigated the association between the serum concentrations of CTRC, OSM, and MMP-10 during exacerbations and those of the other cardiovascular biomarkers listed in Supplementary Table S1. While the serum concentration of CTRC only displayed a strong correlation with brother of CDO (BOC; Figure 5A), the serum concentration of OSM displayed strong and positive correlations with six other cardiovascular biomarkers (Figure 5B–G): interleukin-1 receptor antagonist (IL-1ra), lectin-type oxidized low-density lipoprotein receptor 1 (LOX-1), IL-6, pentraxin 3 (PTX3), chemokine ligand 1 (CXCL1), and carcinoembryonic antigen-related cell adhesion molecule 8 (CEACAM8). In contrast, the serum concentration of MMP-10 during exacerbation failed to display any statistically significant correlation with those of other cardiovascular markers tested. (not shown).

### Higher serum concentration of MMP-10 during stable disease is associated with a higher number of exacerbations and higher blood eosinophil counts.

**Figure 6:**
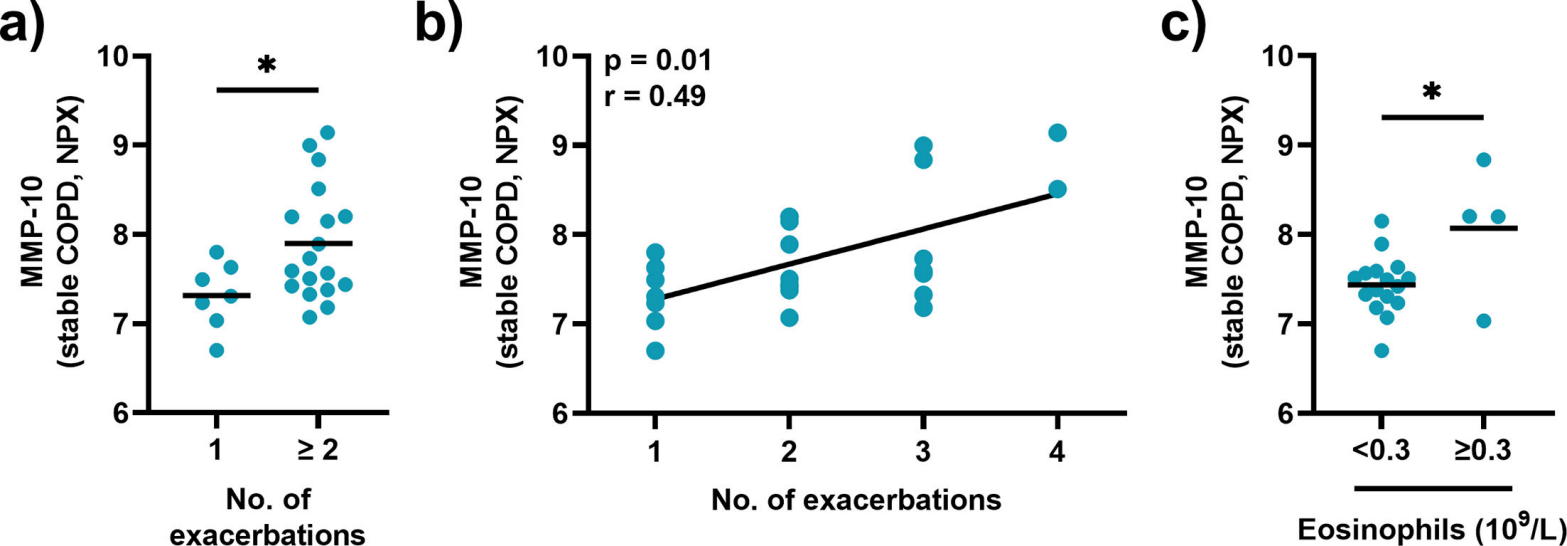
Association of MMP-10 and the number of exacerbations in patients with COPD and CB. Concentrations of MMP-10 quantified in serum samples from patients during stable disease. (**a**) Comparison of MMP-10 levels between patients who experienced 1 or more exacerbations, respectively, during the study. (**b**) Spearman correlation analysis of the protein levels of MMP-10 during stable disease and the number of exacerbations experienced during the study. (**c**) Comparison of MMP-10 levels between patients who had<0·3 × 10^9^ /L and≥0·3 × 10^9^ /L eosinophils, respectively. Closed circles represent individual patients (*n* = 25 in A and B, and *n* = 20 in (**c**). Mean values are indicated by horizontal lines. Statistical significance was determined by unpaired Student’s t-test. *P < 0·05.

We also determined whether the serum concentrations of CTRC, OSM, and MMP-10 measured during stable disease in the COPD-CB group were associated with the number of exacerbations that occurred during the study. Notably, the concentration of MMP-10 was higher during stable disease among patients in the COPD-CB group who had two or more exacerbations compared with those who had one exacerbation only (Figure 6A). Moreover, in this group, the concentration of MMP-10 during stable disease displayed a moderate positive correlation with the number of exacerbations (Figure 6B). In addition, patients with a blood eosinophil count ≥ 0·3 × 10^9^/L, who are known to have a higher risk for COPD exacerbations, also had a higher concentration of MMP-10 during stable disease (Figure 6C). Notably, neither CTRC nor OSM displayed any statistically significant associations or differences regarding the number of exacerbations or blood eosinophil count in the COPD-CB group during stable disease (Supplementary Figure S5).

### Serum concentrations of OSM and MMP-10 during stable disease correlate with blood leukocytes and smoking history, respectively

**Figure 7:**
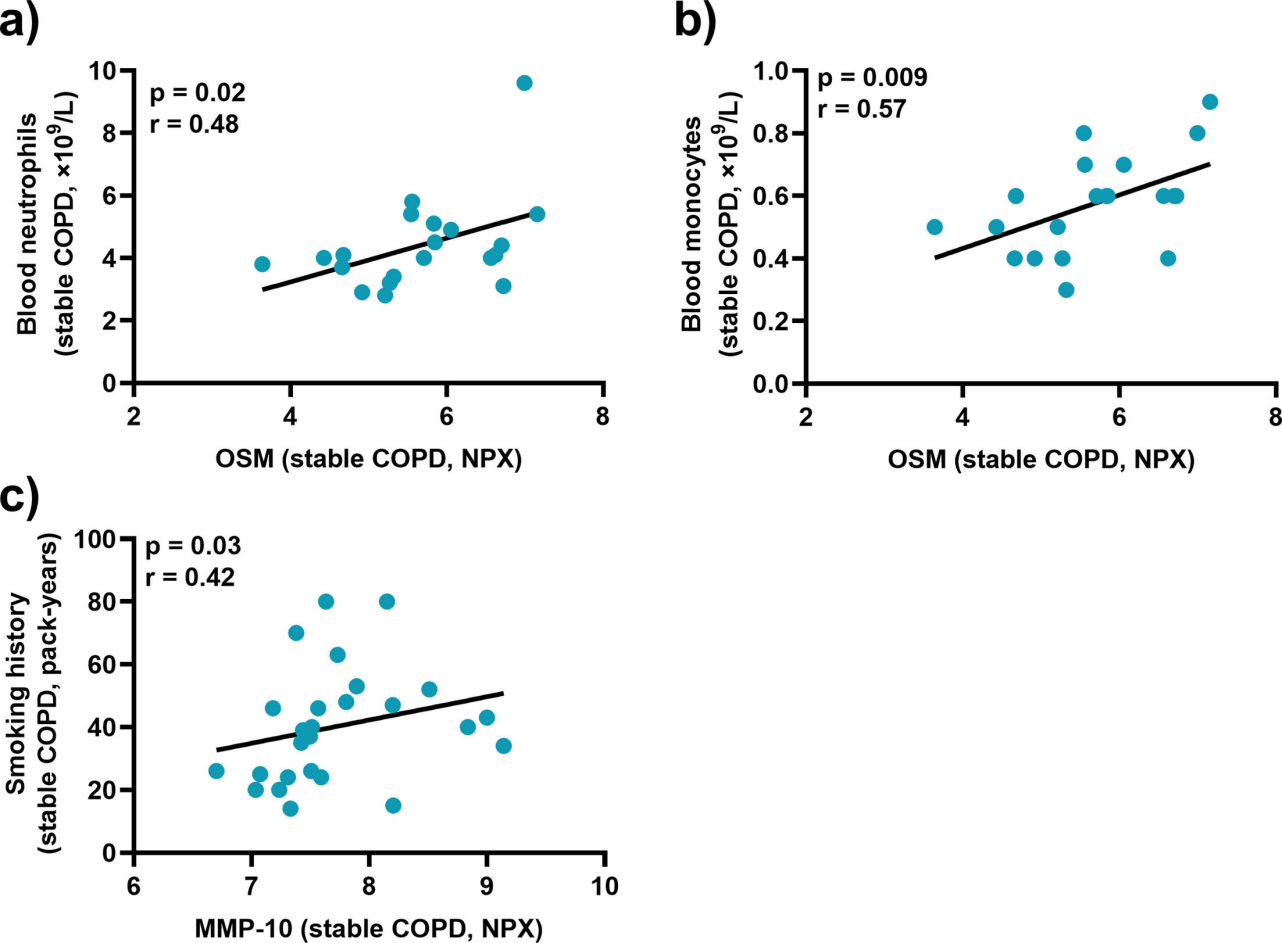
Associations of OSM and MMP-10 with blood leukocytes and smoking history during stable disease in patients with COPD and CB. Spearman correlation analyses of the protein levels of OSM in serum and the (**a**) blood concentration of neutrophils or the (**b**) blood concentration of monocytes, as well as the protein levels of CTRC and (**c**) smoking history, during stable disease. Closed circles represent individual patients (*n* = 20 in A and B, and *n* = 26 in C).

Finally, we assessed the relationship between the serum concentrations of CTRC, OSM, and MMP-10 and the clinical characteristics listed in Table 1 during stable disease. Notably, we found that the concentrations of OSM displayed a positive correlation with those of blood neutrophils and monocytes (Figure 7A and B), while the concentrations of MMP-10 displayed a positive correlation with smoking history (Figure 7C). Nonetheless, we did not detect any statistically significant correlation between the serum concentrations of CTRC, OSM, or MMP-10 and key lung function outcomes (FEV1, FEV1/FVC, and DLCO), or the blood concentrations of CRP, lymphocytes, or basophils (not shown).

## Discussion

Among all 177 serum proteins targeted in the current study, only three were substantially altered during exacerbations in the COPD-CB group: CTRC, OSM, and MMP-10. Notably, these three proteins have previously been linked to CVD [20–25], though they have never been investigated in the context of COPD exacerbations. Moreover, we found that the serum concentrations of CTRC and OSM during exacerbations correlated with two aspects of blood oxygen saturation, CRP levels, and blood leukocytes, all of which are used in the clinic to assess the severity of an exacerbation. Additionally, the serum concentrations of CTRC and OSM during exacerbations correlated with multiple other cardiovascular biomarkers. In contrast, we found that the serum concentrations of MMP-10 during stable disease correlated with the total number of exacerbations in the COPD-CB group. Finally, we also found that the serum concentrations of OSM and MMP-10 during stable disease correlated with blood leukocytes and smoking history, while high MMP-10 concentrations were associated with high blood eosinophil counts, thus linking these serum proteins to known risk factors in COPD. Nevertheless, the serum concentrations of CTRC, OSM, and MMP-10 during stable disease in the COPD-CB group were similar to those of the control groups. Of note, it is important to emphasize that all exacerbation samples were taken upon arrival at the clinic and prior to emergency treatment. Therefore, it is possible that samples collected after treatment or at later time points might have yielded different results.

Chymotrypsin C (CTRC), also known as caldecrin or elastase 4, is a protease produced by pancreatic acinar cells that can degrade trypsinogen and trypsin [26] and has potent calcium-decreasing (hypocalcemic) properties in blood [27]. Importantly, reduced production of CTRC is associated with a number of cardiovascular, respiratory, and endocrine diseases. For instance, extracellular vesicles isolated from blood of patients who experienced myocardial infarction contain less CTRC than those isolated from blood of healthy controls [20]. In addition, loss-of-function mutations in the *CTRC* gene result in chronic pancreatitis due to unrestrained trypsin activity [28]. Importantly, studies on animal models indicate that unrestrained trypsin activity can also lead to emphysema [29], and thus a decrease in CTRC expression might be relevant for the development of emphysema in patients with COPD. In fact, a previous study demonstrated that expression of the *CTRC* gene is downregulated in patients with severe or very severe COPD (GOLD stages 3–4) when compared to those with milder disease (GOLD stage 2) [30]. In the present study, we show that the serum concentration of CTRC is decreased during exacerbations of COPD, and that this decrease is associated with a sharper decline in blood oxygen levels and a marked increase in the blood levels of CRP, monocytes, and neutrophil elastase—a key enzyme involved in emphysema development. Moreover, we show that a decrease in the serum concentration of CTRC during exacerbations of COPD is associated with a similar decrease in the cardiovascular biomarker BOC, a decrease that has previously been linked to hypoglycemia—a known risk factor for CVD [31]. Given the previously published evidence linking myocardial infarction with reduced CTRC content in blood extracellular vesicles, our current findings on reduced systemic levels of CTRC during exacerbations of COPD are fully compatible with an increased cardiorespiratory risk in this clinical setting.

Oncostatin M (OSM) is a multifunctional cytokine that originally derived its name from its ability to inhibit the proliferation of numerous cancer cell lines [32]. Nevertheless, OSM can be released by leukocytes under different circumstances [33], and an increase in OSM has been associated with cardiorespiratory diseases and severe infections. First, the blood concentration of OSM is increased in patients with heart failure [21]. In this context, OSM can have multifaceted effects: while OSM has a protective effect on the heart immediately after myocardial infarction [22,23], continuous stimulation with OSM can in turn lead to heart failure [23,24]. Second, the concentration of OSM is increased in the airways of patients with stable COPD [34] or severe asthma [35], as well as in the blood of patients with bacterial septicemia [36]. Moreover, a recent study implicates these increased levels of OSM in respiratory exacerbations, given that deletion of the *OSM* gene or treatment with an anti-OSM blocking antibody prevented bacterial-driven exacerbations in a mouse model of allergic asthma [37]. Notably, we now show that the blood concentration of OSM increases during exacerbations in patients with COPD and that this increase is associated with lower blood oxygen levels and higher blood levels of CRP, neutrophils, and myeloperoxidase—an enzyme implicated in both COPD progression and CVD risk [38,39]. In addition, we now show that an increase in the blood concentration of OSM during exacerbations of COPD is linked to an increase in the blood concentration of six other cardiovascular biomarkers: IL-1ra, LOX-1, IL-6, PTX3, CXCL1, and CEACAM8, all of which have been linked to increased risk for CVD [40–45]. Thus, our results further strengthen the association between increased OSM levels and a higher cardiorespiratory risk.

Matrix metalloproteinase 10 (MMP-10), also known as stromelysin-2 or transin-2, is released by macrophages and, to a lesser extent, epithelial cells in most tissues upon injury or infection. Importantly, a study on mice suggests that production of MMP-10 increases in the heart following myocardial infarction [25]. In addition, several lines of evidence implicate MMP-10 in the development of the pulmonary comorbidity emphysema. First, smokers and patients with emphysema have increased concentrations of MMP-10 in blood and bronchoalveolar lavage samples [46,47]. Second, mice lacking the *MMP10* gene fail to develop emphysema even after 6 months of chronic exposure to cigarette smoke [48]. Third, MMP-10 has been identified as a putative driver of alveolar wall remodeling in a large gene-wide association study in patients with COPD [48]. Furthermore, we now complement these findings by showing that the serum concentration of MMP-10 is increased in exacerbations of COPD when compared to stable disease in paired study samples. In contrast, a previous study failed to detect a statistically significant difference in the plasma concentration of MMP-10 during exacerbations of COPD and stable disease in unpaired study samples [49]. However, this discrepancy might be easily explained by a key factor: the patient material used in that study was very small (*n* = 6 patients per group). In addition, it is unclear whether that study considered having allergy as a reason for exclusion, and all their patients with COPD exacerbations had community-acquired pneumonia, two factors that may have confounded their results. Finally, we also show that the serum concentration of MMP-10 during stable disease correlates with a higher number of exacerbations and pack-years, as well as blood eosinophil counts above the established threshold of 0·3 × 10^9^ /L, all of which are risk factors for disease progression [1]. We therefore think that our results are compatible with a positive association between a systemic increase in MMP-10 during exacerbations of COPD and an elevated cardiorespiratory risk.

In conclusion, while previous studies have implicated CTRC, OSM, and MMP-10 in CVD, our study forwards original evidence that the serum concentrations of these proteins bear potential as cardiorespiratory biomarkers in patients with COPD and CB. Collectively, these biomarkers display substantial alterations during exacerbations and associated with the number and severity of exacerbations plus additional risk factors such as elevated blood eosinophils. This novel evidence provides a rationale for future assessment of CTRC, OSM, and MMP-10 in larger prospective studies on cardiorespiratory risk in patients with COPD and CB that include patients with varying exacerbation severity.

**Clinical Perspectives**

- Background:
  COPD patients have a markedly increased risk for cardiovascular disease (CVD), especially during exacerbations. In fact, many COPD patients die from CVD during or shortly after exacerbations. Despite this, there is very limited information on alterations in cardiorespiratory biomarkers during exacerbations.
- Result summary:
  Using a proteomics approach, this study identifies three cardiorespiratory biomarkers (CTRC, OSM, and MMP-10) that are markedly altered during COPD exacerbations. Moreover, these biomarkers associate with the number and severity of exacerbations plus additional risk factors such as elevated blood eosinophils.
- Clinical significance:
  While previous studies have implicated CTRC, OSM, and MMP-10 in cardiovascular disease, our study is the first to implicate them in exacerbations of COPD. Taken together, all the available evidence suggests that these three proteins bear potential as cardiorespiratory biomarkers in patients with COPD and motivates further prospective studies to test their clinical utility.

## Supplementary material

Online supplementary material 1

## Data Availability

Data are available upon reasonable request to corresponding author. European Data Regulations preclude open deposition of sensitive personal data (including proteomics) into public repositories.
