# Supplementary material for: Serum proteomics links the cardiorespiratory biomarkers CTRC, OSM, and MMP-10 to exacerbation severity and number in patients with COPD
Source: Clin Sci (Lond). 2025 May 9;139(9):449–62. doi: 10.1042/CS20255852 (PMC12203990; doi:10.1042/CS20255852)
Supplement: Online supplementary material 1 [file CS-139-09-CS20255852-s001.docx]

**Supplementary Material**

**Supplementary Table 1. Proteins detected by the Olink Target 96 Cardiovascular II panel.**

| 2,4-dienoyl-CoA reductase, mitochondrial (DECR1) | Macrophage metalloelastase (MMP12) |
| --- | --- |
| A disintegrin and metalloproteinase with thrombospondin motifs 13 (ADAMTS13) | Macrophage receptor MARCO (MARCO) |
| Advanced glycosylation end product-specific receptor (AGER) | Matrilysin (MMP7) |
| Agouti-related protein (AGRP) | NF-kappa-B essential modulator (IKBKG) |
| Alpha-L-iduronidase (IDUA) | Natriuretic peptides B (NPPB) |
| Angiopoietin-1 (ANGPT1) | Osteoclast-associated immunoglobulin-like receptor (OSCAR) |
| Angiopoietin-1 receptor (TEK) | Oxidized low-density lipoprotein receptor 1 (OLR1) |
| Angiotensin-converting enzyme 2 (ACE2) | P-selectin glycoprotein ligand 1 (SELPLG) |
| Bone morphogenetic protein 6 (BMP6) | Pappalysin-1 (PAPPA) |
| Brother of CDO (BOC) | Pentraxin-related protein PTX3 (PTX3) |
| C-C motif chemokine 17 (CCL17) | Placenta growth factor (PGF) |
| C-C motif chemokine 3 (CCL3) | Platelet-derived growth factor subunit B (PDGFB) |
| CD40 ligand (CD40LG) | Poly [ADP-ribose] polymerase 1 (PARP1) |
| Carbonic anhydrase 5A, mitochondrial (CA5A) | Polymeric immunoglobulin receptor (PIGR) |
| Carcinoembryonic antigen-related cell adhesion molecule 8 (CEACAM8) | Pro-adrenomedullin (ADM) |
| Cathepsin L1 (CTSL) | Pro-interleukin-16 (IL16) |
| Chymotrypsin-C (CTRC) | Programmed cell death 1 ligand 2 (PDCD1LG2) |
| Cobalamin binding intrinsic factor (CBLIF) | Proheparin-binding EGF-like growth factor (HBEGF) |
| Decorin (DCN) | Prolargin (PRELP) |
| Dickkopf-related protein 1 (DKK1) | Prostasin (PRSS8) |
| Fatty acid-binding protein, intestinal (FABP2) | Protein AMBP (AMBP) |
| Fibroblast growth factor 21 (FGF21) | Protein-glutamine gamma-glutamyltransferase 2 (TGM2) |
| Fibroblast growth factor 23 (FGF23) | Proteinase-activated receptor 1 (F2R) |
| Follistatin (FST) | Proto-oncogene tyrosine-protein kinase Src (SRC) |
| Galectin-9 (LGALS9) | Renin (REN) |
| Gastrotropin (FABP6) | SLAM family member 5 (CD84) |
| Growth-regulated alpha protein (CXCL1) | SLAM family member 7 (SLAMF7) |
| Growth/differentiation factor 2 (GDF2) | Serine protease 27 (PRSS27) |
| Heat shock protein beta-1 (HSPB1) | Serine/threonine-protein kinase 4 (STK4) |
| Heme oxygenase 1 (HMOX1) | Serpin A12 (SERPINA12) |
| Hepatitis A virus cellular receptor 1 (HAVCR1) | Somatotropin (GH1) |
| Hydroxyacid oxidase 1 (HAO1) | Sortilin (SORT1) |
| Integrin beta-1-binding protein 2 (ITGB1BP2) | Spondin-2 (SPON2) |
| Interleukin-1 receptor antagonist protein (IL1RN) | Superoxide dismutase [Mn], mitochondrial (SOD2) |
| Interleukin-1 receptor-like 2 (IL1RL2) | T-cell surface glycoprotein CD4 (CD4) |
| Interleukin-17D (IL17D) | Thrombomodulin (THBD) |
| Interleukin-18 (IL18) | Thrombopoietin (THPO) |
| Interleukin-27 (EBI3_IL27) | Thrombospondin-2 (THBS2) |
| Interleukin-4 receptor subunit alpha (IL4R) | Tissue factor (F3) |
| Interleukin-6 (IL6) | Tumor necrosis factor receptor superfamily member 10A (TNFRSF10A) |
| Kit ligand (KITLG) | Tumor necrosis factor receptor superfamily member 10B (TNFRSF10B) |
| Lactoylglutathione lyase (GLO1) | Tumor necrosis factor receptor superfamily member 11A (TNFRSF11A) |
| Leptin (LEP) | Tumor necrosis factor receptor superfamily member 13B (TNFRSF13B) |
| Lipoprotein lipase (LPL) | Tyrosine-protein kinase Mer (MERTK) |
| Low affinity immunoglobulin gamma Fc region receptor II-b (FCGR2B) | V-set and immunoglobulin domain-containing protein 2 (VSIG2) |
| Lymphotactin (XCL1) | Vascular endothelial growth factor D (VEGFD) |

**Supplementary Table 2. Proteins detected by the Olink Target 96 Inflammation panel.**

| Adenosine deaminase (ADA) | Interleukin-18 receptor 1 (IL18R1) |
| --- | --- |
| Artemin (ARTN) | Interleukin-2 (IL2) |
| Axin-1 (AXIN1) | Interleukin-2 receptor subunit beta (IL2RB) |
| Beta-nerve growth factor (NGF) | Interleukin-20 (IL20) |
| C-C motif chemokine 13 (CCL13) | Interleukin-20 receptor subunit alpha (IL20RA) |
| C-C motif chemokine 19 (CCL19) | Interleukin-22 receptor subunit alpha-1 (IL22RA1) |
| C-C motif chemokine 2 (CCL2) | Interleukin-24 (IL24) |
| C-C motif chemokine 20 (CCL20) | Interleukin-33 (IL33) |
| C-C motif chemokine 23 (CCL23) | Interleukin-4 (IL4) |
| C-C motif chemokine 25 (CCL25) | Interleukin-5 (IL5) |
| C-C motif chemokine 28 (CCL28) | Interleukin-6 (IL6) |
| C-C motif chemokine 3 (CCL3) | Interleukin-7 (IL7) |
| C-C motif chemokine 4 (CCL4) | Interleukin-8 (CXCL8) |
| C-C motif chemokine 7 (CCL7) | Interstitial collagenase (MMP1) |
| C-C motif chemokine 8 (CCL8) | Kit ligand (KITLG) |
| C-X-C motif chemokine 10 (CXCL10) | Leukemia inhibitory factor (LIF) |
| C-X-C motif chemokine 11 (CXCL11) | Leukemia inhibitory factor receptor (LIFR) |
| C-X-C motif chemokine 5 (CXCL5) | Lymphotoxin-alpha (LTA) |
| C-X-C motif chemokine 6 (CXCL6) | Macrophage colony-stimulating factor 1 (CSF1) |
| C-X-C motif chemokine 9 (CXCL9) | NAD-dependent protein deacetylase sirtuin-2 (SIRT2) |
| CUB domain-containing protein 1 (CDCP1) | Natural killer cell receptor 2B4 (CD244) |
| Caspase-8 (CASP8) | Neurotrophin-3 (NTF3) |
| Cystatin-D (CST5) | Neurturin (NRTN) |
| Delta and Notch-like epidermal growth factor-related receptor (DNER) | Oncostatin-M (OSM) |
| Eotaxin (CCL11) | Programmed cell death 1 ligand 1 (CD274) |
| Eukaryotic translation initiation factor 4E-binding protein 1 (EIF4EBP1) | Protein S100-A12 (S100A12) |
| Fibroblast growth factor 19 (FGF19) | Protransforming growth factor alpha (TGFA) |
| Fibroblast growth factor 21 (FGF21) | STAM-binding protein (STAMBP) |
| Fibroblast growth factor 23 (FGF23) | Signaling lymphocytic activation molecule (SLAMF1) |
| Fibroblast growth factor 5 (FGF5) | Stromelysin-2 (MMP10) |
| Fms-related tyrosine kinase 3 ligand (FLT3LG) | Sulfotransferase 1A1 (SULT1A1) |
| Fractalkine (CX3CL1) | T-cell differentiation antigen CD6 (CD6) |
| Glial cell line-derived neurotrophic factor (GDNF) | T-cell surface glycoprotein CD5 (CD5) |
| Growth-regulated alpha protein (CXCL1) | T-cell surface glycoprotein CD8 alpha chain (CD8A) |
| Hepatocyte growth factor (HGF) | Thymic stromal lymphopoietin (TSLP) |
| Interferon gamma (IFNG) | Transforming growth factor beta-1 proprotein (TGFB1) |
| Interleukin-1 alpha (IL1A) | Tumor necrosis factor (TNF) |
| Interleukin-10 (IL10) | Tumor necrosis factor ligand superfamily member 10 (TNFSF10) |
| Interleukin-10 receptor subunit alpha (IL10RA) | Tumor necrosis factor ligand superfamily member 11 (TNFSF11) |
| Interleukin-10 receptor subunit beta (IL10RB) | Tumor necrosis factor ligand superfamily member 12 (TNFSF12) |
| Interleukin-12 subunit beta (IL12B) | Tumor necrosis factor ligand superfamily member 14 (TNFSF14) |
| Interleukin-13 (IL13) | Tumor necrosis factor receptor superfamily member 11B (TNFRSF11B) |
| Interleukin-15 receptor subunit alpha (IL15RA) | Tumor necrosis factor receptor superfamily member 5 (CD40) |
| Interleukin-17A (IL17A) | Tumor necrosis factor receptor superfamily member 9 (TNFRSF9) |
| Interleukin-17C (IL17C) | Urokinase-type plasminogen activator (PLAU) |
| Interleukin-18 (IL18) | Vascular endothelial growth factor A (VEGFA) |

**
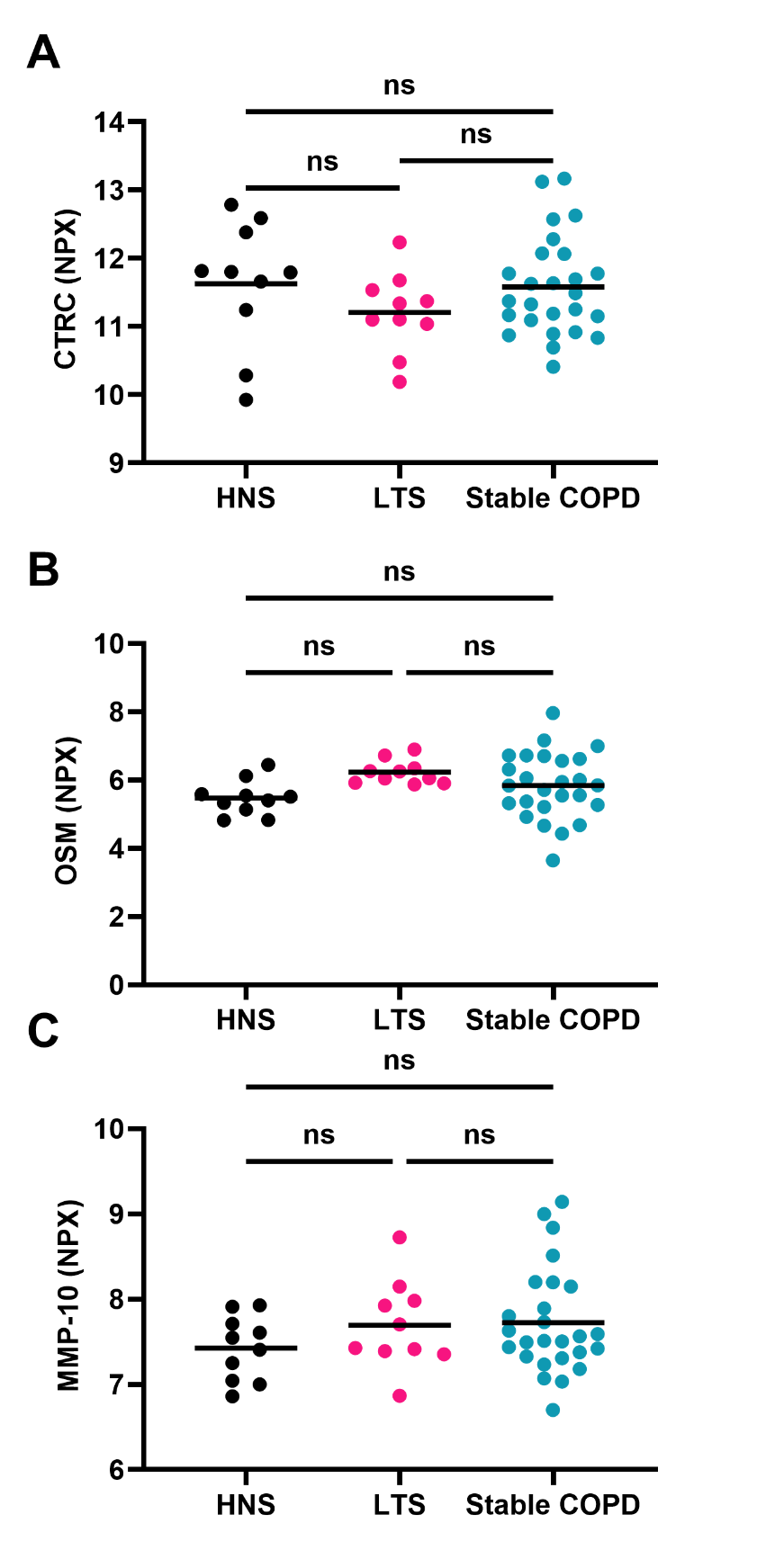
**

**Supplementary Figure 1. The serum concentrations of CTRC, OSM, and MMP-10 are unaltered during stable COPD.** Concentrations of **(A)** CTRC, **(B)** OSM, and **(C)** MMP-10 quantified in serum samples from healthy non-smokers (HNS), long-term smokers (LTS) without COPD, and patients with COPD-CB during stable COPD. Closed circles represent individual patients. Mean values are indicated by horizontal lines. Statistical significance was determined by ANOVA followed by Tukey’s post-hoc test (ns = p ≥ 0.05).

**
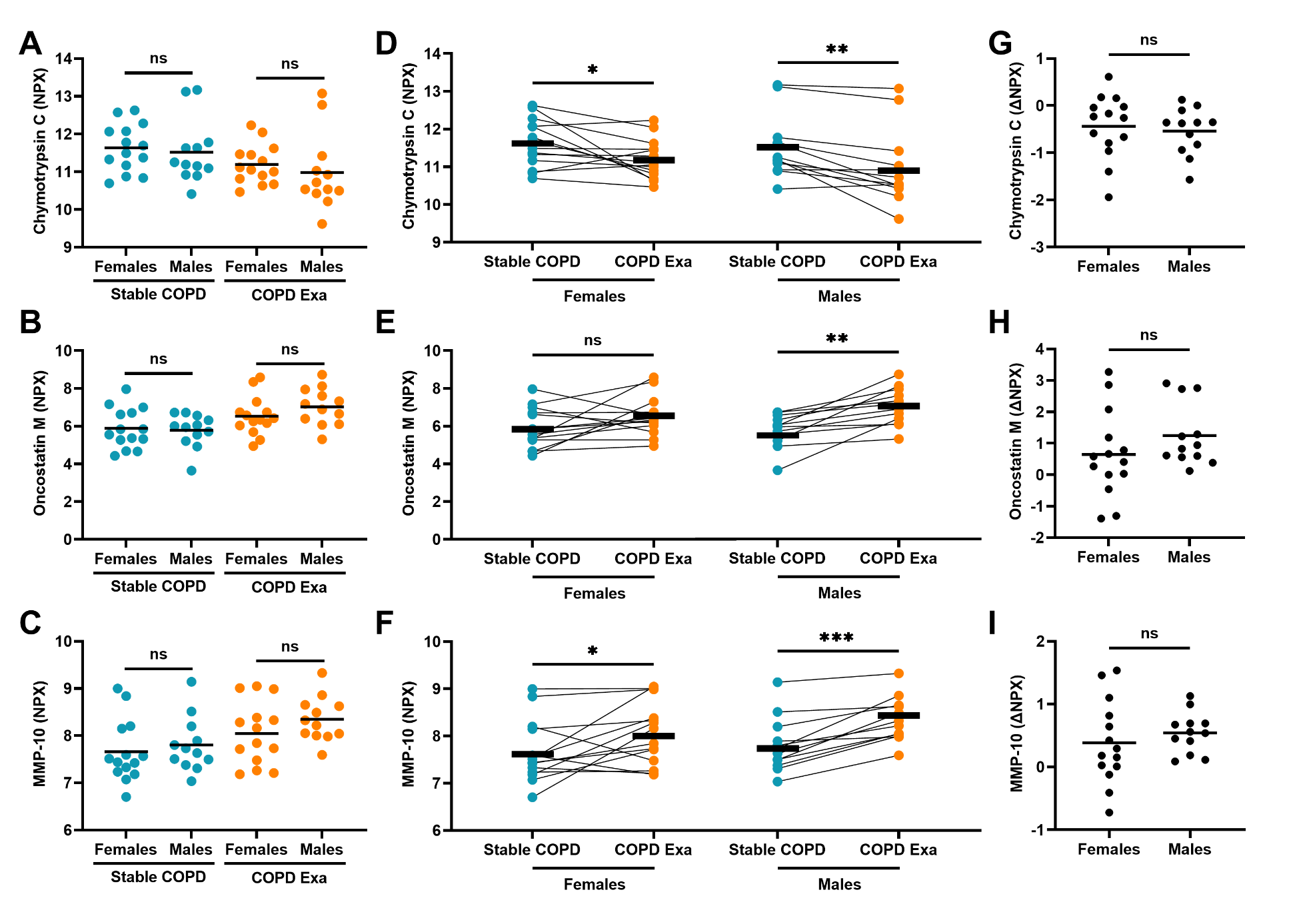
**

**Supplementary Figure 2. There are no sex-related differences in the serum concentrations of CTRC, OSM, and MMP-10 in our patient material. (A-C)** Comparisons between females and males of the serum concentrations of **(A)** CTRC, **(B)** OSM, and **(C)** MMP-10 during stable COPD and an exacerbation (Exa) separately. **(D-F)** Comparisons between stable COPD and a COPD exacerbation of the serum concentrations of **(D)** CTRC, **(E)** OSM, and **(F)** MMP-10 in females and males separately. **(G-I)** Comparisons between females and males of the change (Δ) in the serum concentrations of **(G)** CTRC, **(H)** OSM, and **(I)** MMP-10 from stable COPD to COPD exacerbation. **(A-C and G-I)** Closed circles represent individual patients. **(D-F)** Closed circles joined by a line represent individual patients. Mean values are represented by horizontal lines. Statistical significance was determined by **(A-C and G-I)** unpaired and **(D-F)** paired Student’s t-test. * p < 0.05, ** p < 0.01, *** p < 0.001, ns = p ≥ 0.05.


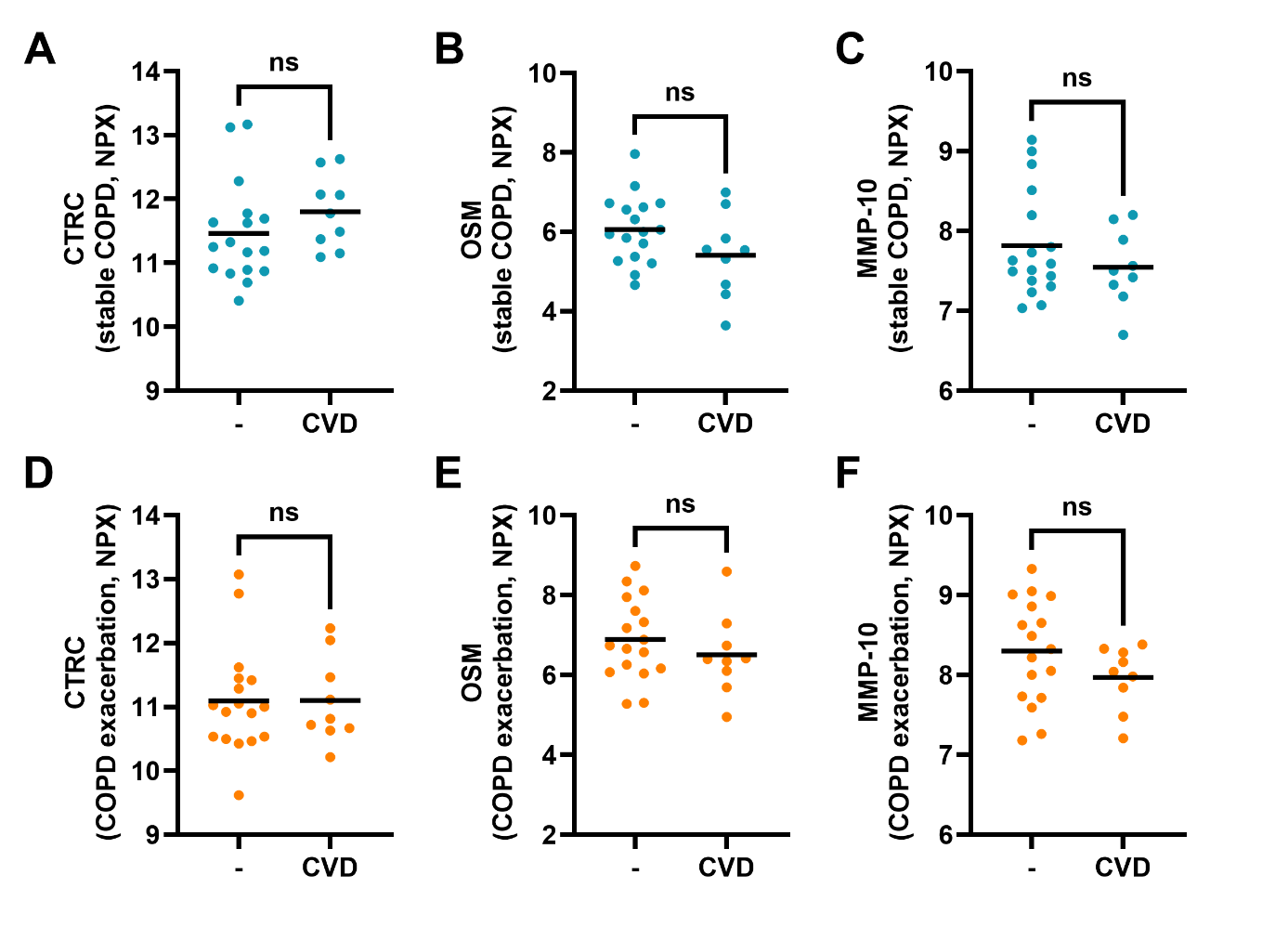


**Supplementary Figure 3. Having a cardiovascular comorbidity at inclusion has no impact on the serum concentrations of CTRC, OSM, and MMP-10.** Comparisons of the serum concentrations of **(A, D)** CTRC, **(B, E)** OSM, and **(C, F)** MMP-10 during **(A-C)** stable clinical conditions and **(D-F)** exacerbation in patients with COPD-CB with and without cardiovascular comorbidities (CVD) at inclusion. Closed circles represent individual patients (n = 26). Mean values are represented by horizontal lines. Statistical significance was determined by unpaired Student’s t-test. Ns = p > 0.05.


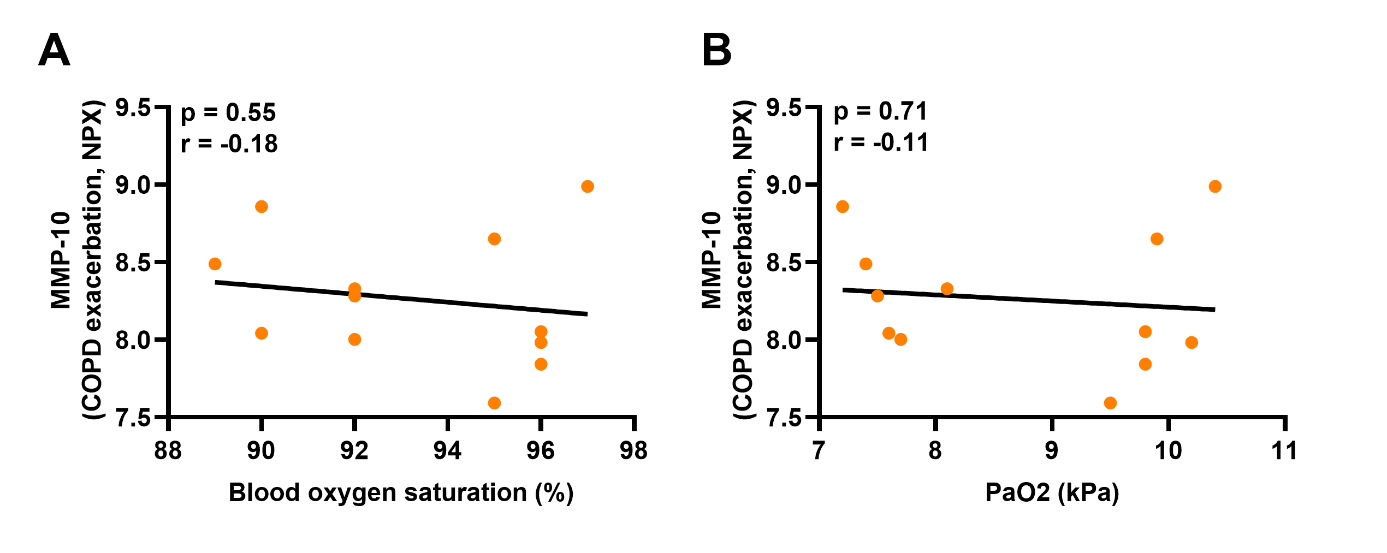


**Supplementary Figure 4. Associations of MMP-10 with blood oxygen saturation and partial pressure of oxygen during a COPD exacerbation.** Spearman correlation analyses of the protein levels of MMP-10 in serum and **(A)** the blood oxygen saturation and **(B)** the arterial partial pressure of oxygen during a COPD exacerbation. Closed circles represent individual patients.


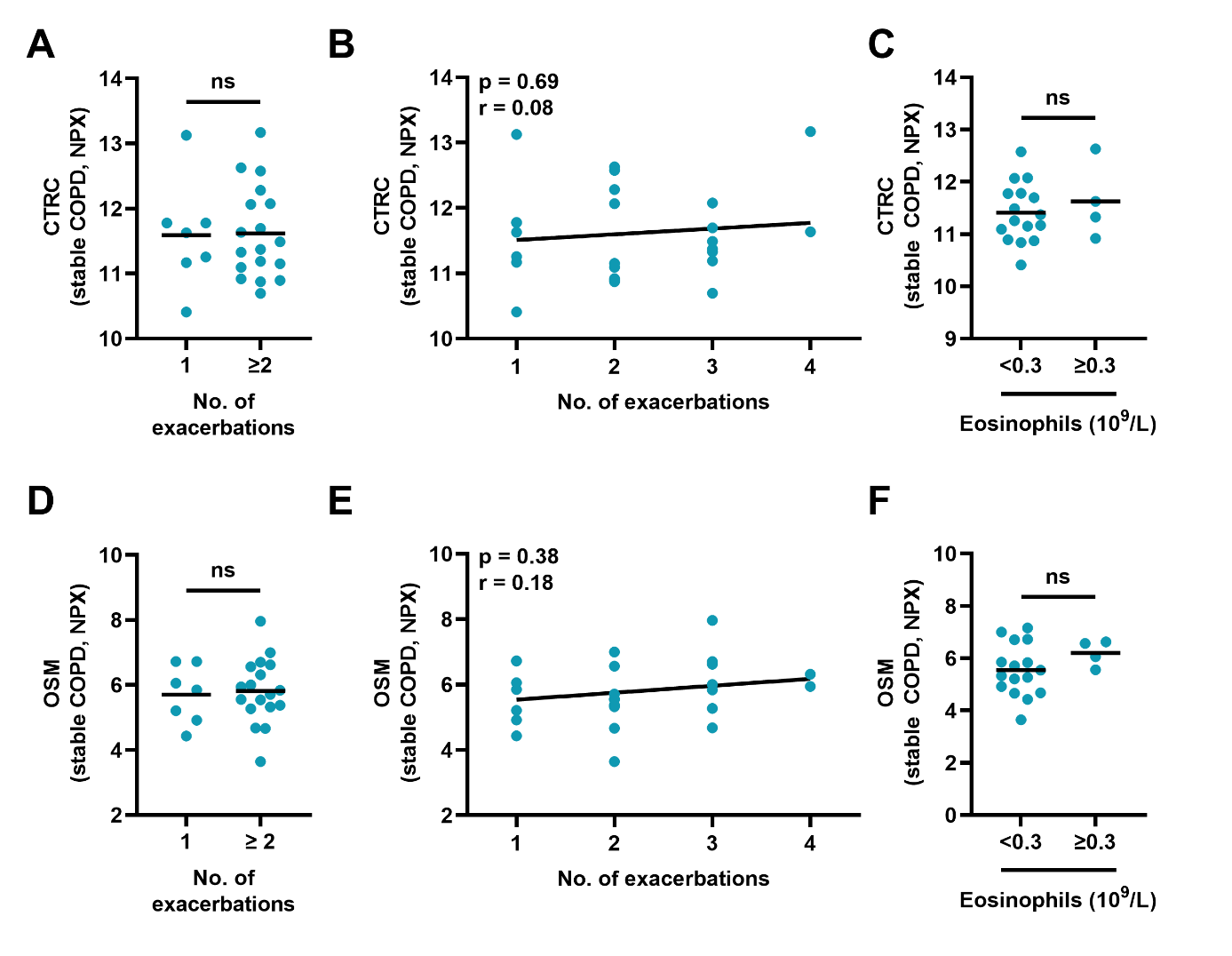


**Supplementary Figure 5. Association of CTRC and OSM and frequency of exacerbations in COPD.** Concentrations of **(A-C)** CTRC and **(D-F)** OSM quantified in serum samples from patients with COPD-CB during stable COPD. Comparison of **(A)** CTRC and **(D)** OSM levels between patients who experienced 1 or more exacerbations, respectively, during the study. Spearman correlation analysis of the protein levels of **(B)** CTRC and **(E)** OSM during stable COPD and number of exacerbations experienced during the study. Comparison of **(C)** CTRC and **(F)** OSM levels between patients who had a low (<0.3×10^9^/L) and a high (≥0.3×10^9^/L) eosinophil concentration, respectively. Closed circles represent individual patients. Mean values are indicated by horizontal lines. Statistical significance was determined by unpaired Student’s t-test (ns = p ≥ 0.05).
